# The groundnut improvement network for Africa (GINA) germplasm collection: a unique genetic resource for breeding and gene discovery

**DOI:** 10.1093/g3journal/jkad244

**Published:** 2023-10-24

**Authors:** Soukeye Conde, Jean-François Rami, David K Okello, Aissatou Sambou, Amade Muitia, Richard Oteng-Frimpong, Lutangu Makweti, Dramane Sako, Issa Faye, Justus Chintu, Adama M Coulibaly, Amos Miningou, James Y Asibuo, Moumouni Konate, Essohouna M Banla, Maguette Seye, Yvette R Djiboune, Hodo-Abalo Tossim, Samba N Sylla, David Hoisington, Josh Clevenger, Ye Chu, Shyam Tallury, Peggy Ozias-Akins, Daniel Fonceka

**Affiliations:** ISRA, Centre d’Etudes Régional pour l’Amélioration de l’Adaptation à la Sécheresse, CERAAS-Route de Khombole, Thiès BP 3320, Senegal; UMR AGAP, CIRAD, 34398 Montpellier, France; CIRAD, INRAE, AGAP, University Montpellier, Institut Agro, 34398 Montpellier, France; F.S.T., Département de B.V., Université Cheikh Anta Diop, BP 5005 Dakar, Senegal; UMR AGAP, CIRAD, 34398 Montpellier, France; CIRAD, INRAE, AGAP, University Montpellier, Institut Agro, 34398 Montpellier, France; National Semi-Arid Resources Research Institute-Serere, PO Box 56, Kampala, Uganda; ISRA, Centre d’Etudes Régional pour l’Amélioration de l’Adaptation à la Sécheresse, CERAAS-Route de Khombole, Thiès BP 3320, Senegal; Mozambique Agricultural Research Institute (Instituto de Investigação Agrária de Moçambique), Northeast Zonal Centre, Nampula Research Station, PO Box 1922, Nampula, Mozambique; Groundnut Improvement Program, Council for Scientific and Industrial Research (CSIR)-Savanna Agricultural Research Institute, PO Box 52, Tamale, Ghana; Zambia Agriculture Research Institute (ZARI), PO Box 510089, Chipata, Zambia; Institut d’Economie Rurale (IER), Centre Régional de Recherche Agronomique (CRRA), BP 281 Kayes, Mali; ISRA, Institut Sénégalais de Recherches Agricoles, Centre National de Recherche Agronomique, BP 53 Bambey, Sénégal; Chitedze Agricultural Research Service, PO Box 158, Lilongwe, Malawi; Institut National de Recherche Agronomique du Niger (INRAN), BP 240 Maradi, Niger; INERA, CREAF, 01 BP 476 Ouagadougou 01, Burkina Faso; Council for Scientific and Industrial Research-Crops Research Institute (CSIR-CRI), P.O. Box 3785, Kumasi, Ghana; INERA, DRREA-Ouest, 01 BP 910 Bobo Dioulasso 01, Burkina Faso; Institut Togolais de Recherche Agronomique (ITRA), 13BP267 Lome, Togo; ISRA, Centre d’Etudes Régional pour l’Amélioration de l’Adaptation à la Sécheresse, CERAAS-Route de Khombole, Thiès BP 3320, Senegal; ISRA, Centre d’Etudes Régional pour l’Amélioration de l’Adaptation à la Sécheresse, CERAAS-Route de Khombole, Thiès BP 3320, Senegal; ISRA, Centre d’Etudes Régional pour l’Amélioration de l’Adaptation à la Sécheresse, CERAAS-Route de Khombole, Thiès BP 3320, Senegal; F.S.T., Département de B.V., Université Cheikh Anta Diop, BP 5005 Dakar, Senegal; Feed the Future Innovation Lab for Peanut, College of Agricultural and Environmental Sciences, University of Georgia, Athens, GA 30602, USA; HudsonAlpha Institute for Biotechnology, Huntsville, AL 35806, USA; Institute of Plant Breeding Genetics and Genomics and Department of Horticulture, College of Agricultural and Environmental Sciences, University of Georgia, Tifton, GA 31793, USA; Plant Genetic Resources Conservation Unit, Griffin, GA 30223, USA; Institute of Plant Breeding Genetics and Genomics and Department of Horticulture, College of Agricultural and Environmental Sciences, University of Georgia, Tifton, GA 31793, USA; ISRA, Centre d’Etudes Régional pour l’Amélioration de l’Adaptation à la Sécheresse, CERAAS-Route de Khombole, Thiès BP 3320, Senegal; UMR AGAP, CIRAD, 34398 Montpellier, France; CIRAD, INRAE, AGAP, University Montpellier, Institut Agro, 34398 Montpellier, France

**Keywords:** groundnut improvement, breeding, germplasm diversity, genotyping, core collection, network, Plant Genetics and Genomics

## Abstract

Cultivated peanut or groundnut (*Arachis hypogaea* L.) is a grain legume grown in many developing countries by smallholder farmers for food, feed, and/or income. The speciation of the cultivated species, that involved polyploidization followed by domestication, greatly reduced its variability at the DNA level. Mobilizing peanut diversity is a prerequisite for any breeding program for overcoming the main constraints that plague production and for increasing yield in farmer fields. In this study, the Groundnut Improvement Network for Africa assembled a collection of 1,049 peanut breeding lines, varieties, and landraces from 9 countries in Africa. The collection was genotyped with the Axiom_Arachis2 48K SNP array and 8,229 polymorphic single nucleotide polymorphism (SNP) markers were used to analyze the genetic structure of this collection and quantify the level of genetic diversity in each breeding program. A supervised model was developed using *dapc* to unambiguously assign 542, 35, and 172 genotypes to the Spanish, Valencia, and Virginia market types, respectively. Distance-based clustering of the collection showed a clear grouping structure according to subspecies and market types, with 73% of the genotypes classified as *fastigiata* and 27% as *hypogaea* subspecies. Using *STRUCTURE*, the global structuration was confirmed and showed that, at a minimum membership of 0.8, 76% of the varieties that were not assigned by *dapc* were actually admixed. This was particularly the case of most of the genotype of the Valencia subgroup that exhibited admixed genetic heritage. The results also showed that the geographic origin (i.e. East, Southern, and West Africa) did not strongly explain the genetic structure. The gene diversity managed by each breeding program, measured by the expected heterozygosity, ranged from 0.25 to 0.39, with the Niger breeding program having the lowest diversity mainly because only lines that belong to the *fastigiata* subspecies are used in this program. Finally, we developed a core collection composed of 300 accessions based on breeding traits and genetic diversity. This collection, which is composed of 205 genotypes of *fastigiata* subspecies (158 Spanish and 47 Valencia) and 95 genotypes of *hypogaea* subspecies (all Virginia), improves the genetic diversity of each individual breeding program and is, therefore, a unique resource for allele mining and breeding.

## Teaser

Speciation of cultivated peanut has greatly reduced its genetic diversity, leading to challenges with crop yield. The Groundnut Improvement Network in Africa (GINA) assemble a collection of 1049 peanut breeding lines, varieties, and landraces from nine African countries and use a high-density SNP array to analyze the genetic structure of the collection and quantify levels of genetic diversity. The authors develop a core collection of 300 lines based on breeding traits and genetic diversity, sharing it as a resource for breeding and discovery purposes.

## Introduction

Peanut or groundnut (*Arachis hypogaea* L.) is a native South American grain legume that is grown in tropical and subtropical regions of the world, mainly by smallholder farmers in Africa and Asia, for food, feed, and income generation. Peanut is consumed by humans as whole nuts and/or as a finished product (e.g. oil, butter, paste, flour, and confectionery) and by animals as haulms and cake (Meyer et al. 2022).

Cultivated peanut is a recent allotetraploid arising from the hybridization of 2 wild diploid species: *A. duranensis* (A genome) and *A. ipaensis* (B genome) followed by chromosome doubling (Bertioli et al. 2016). The speciation of cultivated peanut, superimposed with domestication, has greatly narrowed its genetic base. Nevertheless, the evolutionary forces such as mutation, recombination between homologous, and homeologous genomes as well as genetic drift created the diversity that has been used to classify cultivated peanut into 2 subspecies (*hypogaea* and *fastigiata*), 6 botanical varieties (*hypogaea*, *hirsuta*, *fastigiata*, *vulgaris*, *aequatoriana*, and *peruviana*), and 3 major market types (Virginia, Spanish, and Valencia) (Krapovickas and Gregory 1994; Bertioli et al. 2011).

Plant breeding is a major lever for improving world food security (Fu 2015). Plant breeding aims to combine as many desirable alleles as possible for traits of interest in order to produce superior cultivars that meet the needs of end-users. Thus, genetic diversity is the foundation of any breeding program. The success of breeding programs is based upon identifying and incorporating genetic diversity from various genetic stocks including elite cultivars, landraces, wild species, etc. (Swarup et al. 2021). In this perspective, quantifying the level of genetic diversity that exists in breeding programs to better guide breeder choice and defining sets of germplasm such as core-collections that maximize this diversity is of paramount importance for increasing crop improvement efficiency.

Core collections are valuable resources for breeding and gene discovery (Brown 1989). Core collections have been developed for several important crop species, including rice, wheat, peanut and sorghum (Grenier et al. 2001; Hao et al. 2006; Zhang et al. 2011; Jiang et al. 2014). They can be developed on a geographical basis (world, continent, regions within continent) (Yang et al. 2011) and/or on species (wild vs cultivated), subspecies, or marker type information (Deu et al. 2006; Dwivedi et al. 2008; Mourad et al. 2020). Core collections of various sample size have been developed for peanut. Holbrook et al. (1993) used passport and morphological data available on the Gerplasm Resources Information Network (GRIN) database for developing a core-collection of 831 accessions from the US peanut germplasm collection. A large core collection of 1,704 accessions that represented 10% of the total peanut genebank collection of 14,310 accessions was developed by the International Crops Research Institute for the Semi-Arid Tropics (ICRISAT) (Upadhyaya et al. 2003). Peanut core-collections of more reduced size, called mini core-collections, that ease their phenotyping were also developed by sampling based on passport and phenotypic data of existing larger collections at ICRISAT (Upadhyaya et al. 2002), in the USA (Holbrook and Dong 2005), and in China (Jiang et al. 2010).

Most of the peanut core-collections were constructed before the sequencing of the peanut genomes and the availability of high throughput genotyping technologies, hence did not result from an exhaustive characterization of the molecular diversity. Moreover, the peanut core-collections were mainly derived from genebank accessions that have the advantage of maximizing diversity for trait discovery but with lower likelihood of quick development of best-performing material when crossed with elite lines.

In this study, we describe the genetic diversity managed by 10 breeding programs in East, Southern, and West Africa. We hypothesized that peanut breeders from different countries each manage small collections of the useful diversity that exist in Africa which, when put together, would represent a unique genetic resource that could be used to map traits of interest and add value to breeding programs. We developed a core collection of 300 genotypes based on breeders’ knowledge of their material and on the molecular marker diversity. The value of the core-collection for the breeding programs is discussed.

## Materials and methods

### Plant materials

#### Assembling the African germplasm collection

**Fig. 1.**
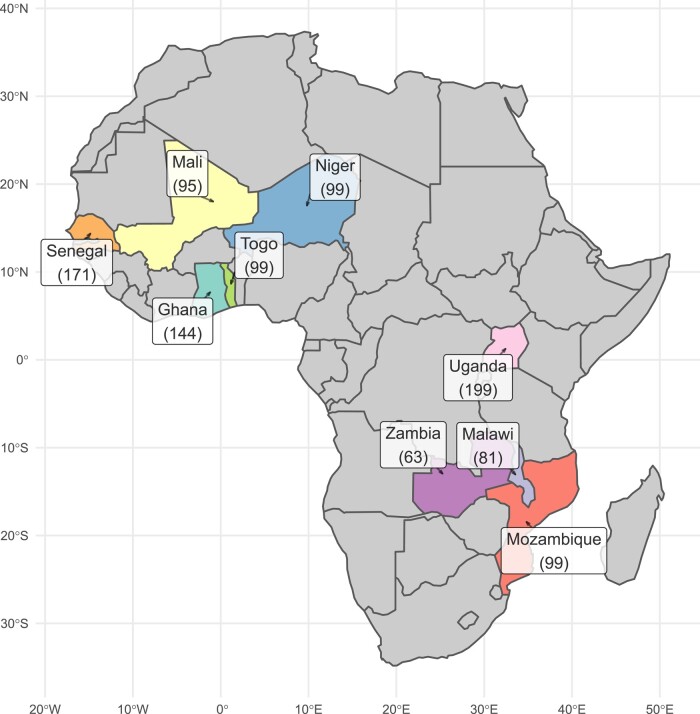
Map of origin of the African germplasm collection. Numbers in brackets are the number of varieties contributed by each country.

A collection of 1,049 groundnut breeding germplasms was assembled from 10 peanut breeding programs located in 9 countries in East, Southern, and West Africa (Table 1 and Fig. 1). The list of varieties along with the information provided by the breeders is presented in Supplementary Table 1.

**Table 1.** Number of varieties contributed by each breeding program in the 9 countries.

Country	Institute	#Varieties
Ghana (Gh1)	Savanna Agricultural Research Institute (CSIR-SARI)	72
Ghana (Gh2)	Crop Research Institute (CSIR-CRI)	72
Malawi (Mlw)	Department of Agricultural Research Services (DARS)	81
Mali (Ml)	Institute of Rural Economy (IER)	94
Mozambique (Mz)	Institute of Agricultural Research (IIAM)	99
Niger (Ng)	National Institute of Agronomic Research of Niger (INRAN)	99
Senegal (Sn)	Senegalese Institute of Agricultural Research (ISRA)	171
Togo (Tg)	Togolese Institute of Agronomic Research (ITRA)	99
Uganda (Ug)	National Semi-Arid Resources Research Institute (NaSARRI)	199
Zambia (Zam)	Zambian Agricultural Research Institute (ZARI)	63
**Total**		1,049

#### Seed multiplication and DNA extraction

All 1,049 genotypes of the collection were grown in the greenhouse of the Centre Régional pour l’Amélioration de l’Adaptation à la Sécheresse (CERAAS), in Thies, Senegal. A single plant was grown for each genotype. DNAs were extracted from dried leaves of 20-day-old plants, using the MATLAB protocol (Risterucci et al. 2000) and purified using the Macherey-Nagel 96 Nucelo rapid ultrafiltration kit.

Seeds were harvested on the same single genotype and stored in the cold-room at CERAAS for further multiplication, use, and sharing.

### Genotypic data

Genotyping was performed using the Affymetrix Axiom_Arachis2 SNP array (Clevenger et al. 2018; Korani et al. 2019). Raw genotyping data were analyzed and filtered using Axiom Analysis Suite (Thermo Fisher). Out of 48,000 SNPs, 8,911 were kept as polymorphic highly reliable markers. The genotyping data were encoded in the Variant Call Format (VCF) (Danecek et al. 2011) and imported into the Gigwa genotypic data management system (Sempéré et al. 2016) deployed on the PeanutBase (Dash et al. 2016) portal at https://www.peanutbase.org/gigwa/. Among these 8,911 SNPs, 6,205 had less than 5% of individuals being scored as heterozygous, which corresponds to what is expected with breeding lines in an autogamous species. When checking thoroughly the segregation profile of the 2,706 markers that had more than 5% of heterozygotes, for 2,224 of them, instead of the expected 3 genotype classes, only 2 genotype classes were observed with one being called as heterozygous. These SNPs corresponded to features on the array that detect loci in both subgenomes and for which apparent heterozygous are homozygous for 1 allele in 1 subgenome and for the alternate allele in the other subgenome resulting in signal from both alleles. For those particular markers (2,224 SNPs) the heterozygous class was thus converted to the alternate homozygous class to reflect the polymorphism of only 1 subgenome. The markers with more than 5% heterozygotes that still exhibited 3 genotypic classes were discarded. The final genotypic dataset included 8,229 SNPs and 1,049 individuals.

### Genetic diversity analysis

#### Assignment of subspecies and market types information

The information provided by the breeders on the subspecies origin and the market type of the lines they nominated was sparse and heterogeneous. In addition, as the varieties used in the different programs could have an external origin, the associated-information might be lost or error prone. For clearly assigning the lines to subspecies and market types, a dataset of 2,209 accessions from the United States Departement of Agriculture (USDA) collection genotyped with the same Axiom_Arachis2 SNP array was used together with available phenotypic observation data for these accessions downloaded from Grin Global (https://npgsweb.ars-grin.gov/gringlobal). The observations included presence/absence of flowers on the main axis, pod type, and pod shape. Out of 2,209 accessions, 625 had congruent observation data on the 3 variables that allowed the unambiguously assignment to *fastigiata* or *hypogaea* subspecies and to Valencia, Spanish, or Virginia market types as outlined in Table 2.

**Table 2.** Number of accessions from USDA collection with congruent observations on presence/absence of flowers on the main axis, pod type, and pod shape allowing to unambiguously assign them to subspecies and market types categories.

Pod shape	Main axis flower	Pod type	Subspecies	Market type	Number of accessions
Fastigiata, Vulgaris, Peruviana	Yes	Valencia	*fastigiata*	Valencia	92
		Spanish		Spanish	155
Hypogaea, Hirsuta	No	Virginia	*hypogaea*	Virginia	378
Total					625

The *dapc* method implemented in the “adegenet” RStudio package (Jombart 2008) was used to assign the genotypes of the African germplasm collection to a subspecies and a market type using the 625 accessions from the USDA collection as a calibration set. To estimate the precision of the *dapc* prediction model, random calibration and validation sets of 500 and 125 accessions respectively were used. A model built on the calibration set was used to predict the validation set and prediction accuracy was estimated from the confusion matrix. A model was then built on the whole set of 625 USDA accessions to predict all the varieties of the African germplasm collection.

#### Collection genetic structure

##### Distance based

Genetic distances were computed using the bitwise.dist function of the poppr R package (Kamvar et al. 2014). A hierarchical clustering tree was computed from genetic distances with Ward's minimum variance method using the hclust function and the ward.D2 parameter (Ward 1963; Murtagh and Legendre 2014). The tree together with different layers of information was represented using the ggtree R package (Yu et al. 2017). Seven large clusters of very closely related material were identified by cutting the hierarchical clustering tree at d = 15 and retaining the clusters having a size equal to or greater to 10.

##### Structure

The model-based approach implemented in the software STRUCTURE v 2.3.4 (Pritchard et al. 2000) was used to infer population structure in the collection. Ten runs with a number of clusters (K) ranging from 2 to 8, a burn-in period of 50.000 steps and 100.000 Monte Carlo Markov Chain (MCMC) replicates were done. Genotypes were assigned to structure groups at a minimum membership of 0.8. Genotypes with a maximum membership probability lower than 0.8 were assigned to an “Admixed” group.

##### Principal component analysis

Principal component analysis (PCA) was performed using the SNPrelate R-package (Zheng et al. 2012). For each of the 7 clusters of very closely related varieties identified in the tree, only 1 member was kept in the PCA analysis. The other members of each cluster were projected as supplementary individuals on the principal components.

#### Within countries/institutes diversity

Expected heterozygosity (*He*) was calculated using the dartR R-package (Gruber et al. 2018) according to the following formula:

$He=1\text{–}(p^{2}+q^{2})$ , where p is the frequency of the reference allele and q is the frequency of the alternative allele.

### Development of a core collection

A core collection of 300 individuals was constructed using a 3-step approach.

- First, breeders nominated 10–15 preferred lines from their breeding program. This first set was considered as trait-based diversity.
- Second, a tree was constructed using the trait-based diversity set and inspected manually for possible closely related lines for which only one was kept.
- Third, the average entry-to-nearest-entry distance (AN) optimization objective and the Modified Roger's (MR) distance of the corehunter3 software (De Beukelaer et al. 2018) were used to select more lines for increasing the diversity of the trait-based set and for representing the whole collection.

## Results

### Composition of the African collection

#### Origin of the genotypes

From only the genotype names provided by the different breeding programs, several duplicates were indicated. Indeed, 99 genotypes were present more than once in the collection, from which 7 were present more than twice (Table 3). For example, 55–437 was nominated 5 times by 5 different programs, Fleur11 4 times, 9 genotypes were nominated 3 times, and 88 genotypes were nominated twice. Sometimes the same genotype was nominated 2 or 3 times by the same breeding program (e.g. ICG12991xCG7 by Mozambique).

**Table 3.** List of genotypes that were nominated more than twice in the African germplasm collection. N = number of nominations.

Genotypes	N	Countries
55–437	5	Ng, Sn, Gh1, Gh2, Ml
Fleur11	4	Ng, Sn, Gh1, Ml
ICGV-00350	3	Tg, Gh2, Ml
ICGV-86015	3	Gh1, Gh2, Ml
ICGV-IS-13827	3	Gh1, Ml, Ml
ICGV-SM01513xJL-24	3	Mz, Mz, Mz
ICG12991xCG-7	3	Mz, Mz, Mz

Out of 1,049 genotypes, 459 had an “ICG” name indicating an origin from ICRISAT: 34 ICG, 120 ICGV, 112 ICGV-IS, 160 ICGV-SM, 1 ICG-SM, and 32 hybrid forms. The proportion of ICG material in the breeding programs ranged from 1% in Senegal to 86% in Mali with an average of 48%.

In Senegal, 114 closely related varieties were labeled as “Precol” with numbers ranging from Precol-2 to Precol-127, which were all derived from interspecific crosses. It was also worth noting the presence in the collection of 9 “12_CS” lines, 4 from Zambia and 5 from Mali. These varieties were introgression lines that are part of an interspecific chromosome segment substitution lines library developed by Fonceka et al. (2012) and distributed in several countries in Africa. Six varieties registered in Senegal and included in the collection were also derived from the same population: Rafeet Kaar, Raw Gadu, Tosset, Yaakar, KomKom, and Jaambar.

#### Assignment of subspecies and market types

As passport data of the African collection were sparse and error prone, an independent dataset of 2,209 accessions from the USDA collection genotyped with the same Axiom_Arachis2 SNP array was used to build a model for predicting subspecies and market type assignment of the genotypes. To test the validity of this approach, a validation set of 125 USDA accessions was predicted using a *dapc* model developed on a calibration set of 500 USDA accessions. The confusion matrix obtained with this procedure showed an accuracy of 93% predicting market type based on SNP data (Table 4).

**Table 4.** Confusion matrix obtained on the prediction of the validation set (125 USDA accessions) using a *dapc* model developed on a calibration set of 500 USDA accessions.

	Spanish	Valencia	Virginia
Spanish	17	0	6
Valencia	1	22	0
Virginia	1	1	77
*Accuracy = 93%*			

**Fig. 2.**
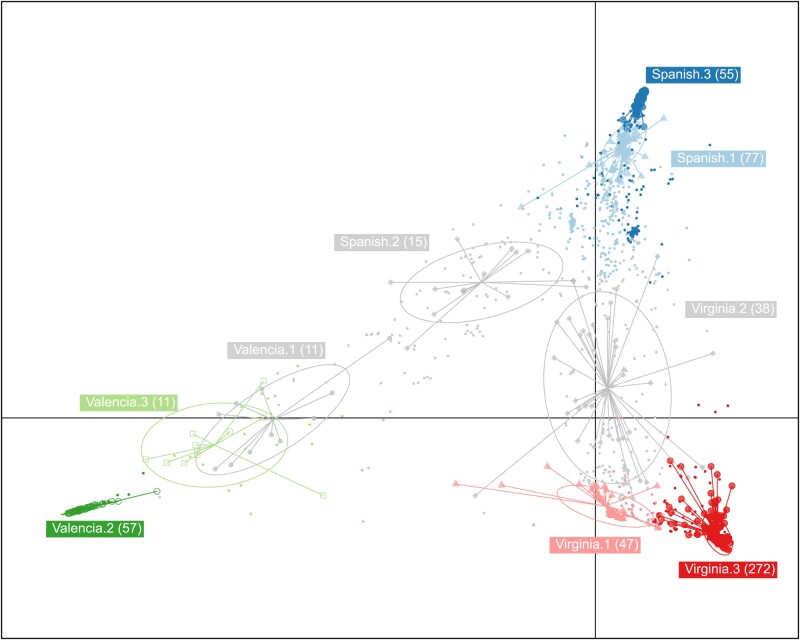
*Dapc* analysis of the 625 USDA accessions based on market type grouping factor. The market type groups were further divided into 3 subgroups by k-means clustering of the PCA space.

After validating the prediction model, 42 outliers that were not accurately predicted were removed from the initial set. Each of the initial market type prior groups were further divided into 3 subgroups by k-means clustering of the PCA space and the 9 resulting groups (Spanish.1,2,3; Valencia.1,2,3; and Virginia.1,2,3) were used as grouping factors of the *dapc* analysis to predict the 1,049 genotypes of the African collection. The varieties assigned to subgroups Spanish.2, Valencia.1 and Viginia.2 that were not well explained by the first discriminant components (Fig. 2) as well as those that had a posterior membership inferior to 0.8 were considered as uncertain (Table 5).

**Table 5.** Assignment of the 1049 varieties of the African germplasm collection to market type group and subspecies using *dapc*.

dapc group	dapc prediction	Final assignment		Subspecies	
Spanish.1	338	326	542	*fastigiata*	577
Spanish.2	92	0			
Spanish.3	222	216			
Valencia.1	10	0	35		
Valencia.2	12	11			
Valencia.3	26	24			
Virginia.1	73	70	172	*hypogaea*	172
Virginia.2	171	0			
Virginia.3	104	102			
Unassigned	1	300			

Among the 1,049 genotypes, 749 were assigned to a market type following this procedure and 300 remained nonassigned (Table 5). Among the assigned varieties, 43.5%, 28.8, 1.4, and 3.2% were from Spanish.1, Spanish.3, Valencia.2, and Valencia.3 subgroups (*fastigiata* subspecies), respectively. Virginia.1 and Virginia.3 subgroups (*hypogaea* subspecies) represented 9.3 and 13.6% of the assigned varieties.

### Collection genetic structure

**Fig. 3.**
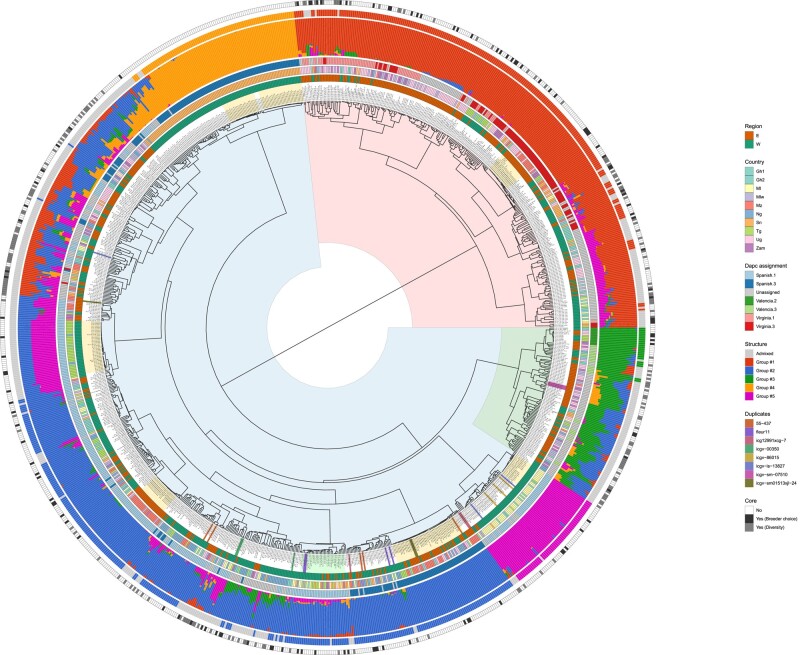
Ward hierarchical clustering tree of the African breeders’ germplasm collection. Six layers of information are depicted as concentric circles: (1) the region (East Africa (E) or West Africa (W) of provenance of the varieties, (2) the/breeding program (Country) that nominated the variety, (3) the market type group assigned by the *dapc* model, (4) the structure barplot of individual ancestry proportions for the genetic clusters inferred at K = 5, (5) the structure group assigned At a minimum membership of 0.8, and vi- the inclusion of each variety in the core collection following a selection by breeders (Yes Breeder choice) or diversity sampling (Yes Diversity). The pale-yellow highlighted varieties are those that are part of clusters of closely related material. The pale-green highlighted varieties are part of the same interspecific population. Other colors represent varieties that are duplicated 3 or more times.

The genetic structure of the collection was depicted by ward hierarchical clustering based on the Euclidian distance computed from SNP data (Fig. 3). The market type assignment described above was represented on the same figure with the “dapc assignment” factor. The distribution of market types in the hierarchical tree reveals a main structure supported by subspecies *hypogaea* and *fastigiata* with 2 main groups that exclusively contain Virginia or nonassigned totalizing 280 genotypes (27%) on one side and Spanish, Valencia, or nonassigned totalizing 749 genotypes (73%) on the other side.

The region and country factors did not strongly explain the genetic structure of the collection. However, it was found that Virginia types were more frequent in breeding programs from East Africa. In addition, some clusters of varieties were typical of specific breeding programs: 2 clusters of “Precol” varieties from Senegal, a cluster of Spanish varieties from Mali and Niger, and a cluster of Spanish varieties from Togo.

Seven clusters of varieties (highlighted in pale yellow in Fig. 3, and described counter clockwise hereinafter) including some of the country specific ones described above, were composed of very closely related material. The first 2 clusters were exclusively composed of Spanish-like “Precol” varieties from Senegal. The third cluster was mostly composed of Spanish varieties from Togo plus 2 from Niger and 2 from Ghana. The fourth cluster was mostly composed of Spanish varieties from Mozambique plus 1 from Zambia and 1 from Malawi. The fifth cluster was constituted mostly of Spanish varieties from Mozambique, Uganda, and Niger plus 5 varieties from Togo, 4 from Ghana, 3 from Malawi, 1 from Mali, and 1 from Zambia. The sixth cluster was mostly composed of Spanish ICGV varieties from Mali, plus 1 from Togo and 2 from Ghana. Finally, the seventh one was a group of Virginia varieties from Uganda and Mozambique.

The position in the tree of the duplicated varieties listed in Table 3 is highlighted in Fig. 3. The variety 55-437 that was nominated 5 times by 5 different programs was spread in 3 different parts of the tree, suggesting 3 divergent versions of this variety. Considering that this variety was registered in Senegal, we can hypothesize that the 2 versions from Senegal and Niger that are closely related represent the original variety while the 3 other ones from Mali and from the 2 programs of Ghana have deviated due to outcrossing or labeling errors. Similarly, the variety Fleur11 that was nominated 4 times was located in 2 different parts, yet quite close, to the tree. The versions from Senegal and Ghana were almost identical while the 2 versions from Niger and Mali were close to each other. Fleur11 was also a variety registered in Senegal (Mortreuil 1993) and was used as recurrent parent of the CSSL population developed by Fonceka et al. (2012) for which some of the derivatives are also included in the collection and cluster with the versions from Senegal and Ghana, confirming conformity of these 2 samples with the original variety. Six other varieties were present 3 times in the collection: ICG-SM-07510 for which the 3 versions from Zambia, Uganda, and Malawi were closely clustered together; ICGV 00350 for which the 2 versions from Togo and Mali were closely related to each other but the third version from Ghana showed some degree of divergence; ICGV 86015 for which the 3 versions from Ghana and Mali were closed to each other but showed some degree of divergence; ICGV-IS 13827 for which 2 versions from Mali and Ghana were in the same cluster but another 1 from Mali was totally different; and ICG12991xCG-7 and ICGV-SM01513xJL-24 from Mozambique that had both 2 closely related versions and a divergent one.

A group of varieties (highlighted in pale green in Fig. 3) was composed of Fleur11, 6 recently released varieties from Senegal (Rafeet Kaar, Raw Gadu, Tosset, Yaakar, KomKom, Jaambar), and all 12CS numbers (Zam-12CS_060, Zam-12CS_069, Zam-12CS_111, Zam-12CS_121, Mal-12CS_116, Mal-12CS_042, Mal-12CS_114, Mal-12CS_010, Mal-12CS_098). Interestingly, 13 other varieties with different names clustered in the same group (Mal−ICGVIS 13871, Oug−SGV 99046 UG, Sen−PrecoL103, Tog−HG55, Tog−HG11, Tog−HG76, Tog−HG82, Tog−HG13, Tog−HG10, Tog−HG87, Gha−ICGV−IS 13052, Mal−ICGVIS 13079, GhaII−ICGV−IS−13052).

The structure of the collection was also analyzed using the model-based approach of the *STRUCTURE* software. It is worth mentioning that the *dapc* and *STRUCTURE* approaches used in this study are very different by nature. Using the *dapc* method, we applied to our collection a supervised model, trained on a different dataset, with the purpose of assigning to each genotype a market type information that is meaningful to breeders. Using the *STRUCTURE* method, we developed an unsupervised population genetics model for a better understanding of why some genotypes were not assigned to a given market type while they belonged to *hypogaea* or *fastigiata* subspecies.

Because of its population genetics model assumptions, *STRUCTURE* is more suitable for the analysis of natural populations than crops (Campoy et al. 2016). Indeed, the collection used in this study is a composite set of genotypes nominated by breeders that is poorly representative, in terms of structure, of the natural variation of the *Arachis hypogaea* species. Because of that, we retained the number of *STRUCTURE* groups (K) that showed the highest stability over 10 runs. At a value of K = 5, 8 runs out of 10 gave consistent results and the resulting groups globally corresponded to the usual groups that breeders are familiar with (subspecies and market types). The group #1 corresponded to subspecies *hypogaea* and market type Virginia, while groups #2 to #5 corresponded to subspecies *fastigiata*, with groups #2 and #3 corresponding to the market types Spanish and Valencia, respectively. Group #4, identified as Spanish by *dapc* assignment, corresponded to the group of “Precol” varieties that were derived from interspecific crosses. Finally, Group #5 corresponded to a group of closely related material, also identified as spanish by *dapc* assignment mainly composed of ICRISAT lines mostly from Mali and related to the JL-24 variety (Fig. 3). Among the 300 varieties that were not assigned by *dapc*, 227 (76%) appeared as admixed using *STRUCTURE*, at a minimum membership of 0.8. This was, for example, the case of the Virginia genotypes located at the right of group #1 that were admixed with Spanish groups #5, #2, and #4. These were also the cases of a series of Valencia genotypes located below group #3 that appeared as admixed with Spanish groups #2, #4, and #5, and with Virginia group #1, and a series of Spanish genotypes located at the left of group #5 that were admixed with Virginia group #1, Valencia group #2 and Spanish groups #2, #4, and #5 (Fig. 3).

A PCA was performed with the same SNP data. The first 2 principal components explained 34.8 and 6.7% of the total variation, respectively. The varieties were distributed on the first principal plan between 3 poles represented by the market types (Supplementary Fig. 1). The first principal component was clearly separating the *fastigiata* and *hypogaea* subspecies while the second component separated Spanish from Valencia in the *fastigiata* subspecies. Interestingly, many varieties were intermediate between Virginia and Spanish or between Spanish and Valencia, which confirms the observed admixture and reveals the work of genetic mixing achieved by breeders.

### Core collection composition

A core collection of 300 varieties was composed from the whole initial African collection. Each program nominated 10–15 preferred lines to constitute an initial set of trait-based breeder's favorites, then this set was extended to 300 varieties by diversity sampling based on genotypic data. Table 6 indicates the number of nominated varieties and sampled varieties for each program, disaggregated by market type and indicating the contribution of each program to the core collection. The core collection represents, by construction, 29% of the initial African breeder's collection. The contribution of each program in the core collection ranged from 6% (Togo) to 18% (Uganda). For each program, the proportion of varieties of the initial collection that were included in the core collection ranged from 18% (Togo) to 44% (Niger). In terms of subspecies and market type, the core collection is composed of 205 genotypes of *fastigiata* subspecies (158 Spanish and 47 Valencia) and 95 genotypes of *hypogaea* subspecies (all Virginia). The distribution of the core collection in the diversity of the global collection is represented in Fig. 3.

**Table 6.** Composition of the core collection with breeding programs’ contribution and selection method.

Country	Breeder Choice	Diversity	Spanish	Valencia	Virginia	Total
Gh1	9	15	10	2	12	24 (8%)/72 (33%)
Gh2	8	18	14	5	7	26 (9%)/72 (36%)
Mlw	10	22	12	11	9	32 (11%)/81 (40%)
Ml	14	14	22	1	5	28 (9%)/94 (30%)
Mz	10	14	19	1	4	24 (8%)/99 (24%)
Ng	15	29	39	5	0	44 (15%)/99 (44%)
Sn	14	16	12	1	17	30 (10%)/171 (18%)
Tg	12	6	9	3	6	18 (6%)/99 (18%)
Ug	12	44	15	14	27	56 (18%)/199 (28%)
Zam	9	9	6	4	8	18 (6%)/63 (29%)
	114	186	158	47	95	300/1049 (29%)

Breeder choice: number of varieties selected by breeders to be included in the core collection; Diversity: number of varieties selected by the corehunter software. Number of varieties for each market type is deduced from their position in the tree. The first percentage is the proportion of a program in the core collection. The second percentage is the proportion of varieties of a program included in the core collection to the initial number in the whole collection.

### Diversity by institutes

**Fig. 4.**
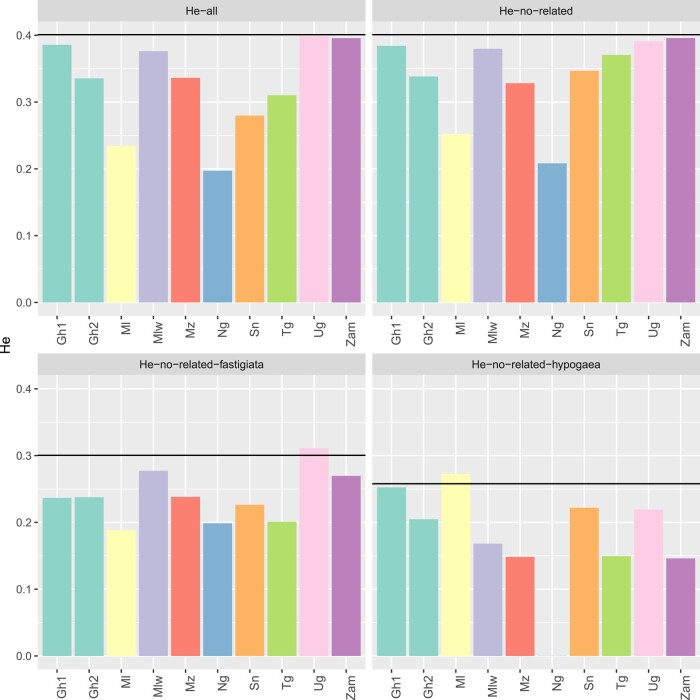
Barplot of expected heterozygosity (he) in different programs. *He-all*: He computed with all genotypes; *He-no-related*: He computed with all genotypes except the closely related ones; *He-no-related-fastigiata*: He computed with genotypes from *fastigiata* subspecies except the closely related ones; *He-no-related-hypogaea*: He computed with genotypes from *hypogaea* subspecies except the closely related ones. The horizontal line represents the He value in the subset of germplasm that belongs to the core collection.

Expected heterozygosity (*He*), as a measure of genetic diversity, was calculated for each country/program in 4 different sets of germplasms (Fig. 4): all genotypes of the program (He-all), all genotypes except the closely related ones described in the previous section (He-no-related), varieties from *fastigiata* subspecies except the closely related ones (He-no-related-fastigiata), and varieties from *hypogaea* subspecies except the closely related ones (He-no-related-hypogaea). *He* was also calculated in the core collection and in the *fastigiata* and *hypogaea* subsets of the core collection. The expected heterozygosity for each program and for the different set of germplasms were compared with each other and with that of the core collection. He-all and He-no-related were higher in Uganda, Zambia, Ghana1, and Malawi and were similar to the diversity of the core-collection, indicating higher genetic diversity managed by the breeding programs of these countries. The lower genetic diversity is observed in Niger mainly because the breeding program in this country used only genotypes from *fastigiata* subspecies. Within countries, genetic diversity was higher for the genotypes belonging to the *fastigiata* subspecies than the *hypogaea* ones. The core-collection contribution to the increase of genetic diversity was important for almost all breeding programs.

## Discussion

### Germplasm exchange and management of the breeding material

Increasing the exchange and use of valuable germplasm in breeding programs is needed to address the global challenge of food security, especially in the face of climate change. This requires collaborative initiatives between multiple actors who are willing to share their genetic resources either in formal or informal networks, with or without explicit negotiated agreements (Louafi and Welch 2021). The Groundnut Improvement Network for Africa is a trans-regional semiformal crop network aimed at enhancing peanut production in Africa through germplasm exchange, characterization, and breeding. In this study, we describe the assembly and the diversity analysis of germplasm managed by 10 breeding programs in 9 countries in East, Southern, and West Africa that are members of this network. Among the nominated breeding germplasms, 48% were traced-back from ICRISAT origin, 2% from USA, 1% from China and probably from many other countries or organizations that could not be identified because of the lack of passport data and the frequent renaming of lines by breeders. Louafi and Welch (2021) reported up to 11% contribution of the Consultative Groupe on International Agricultural Research (CGIAR) genebanks to the germplasm used by national breeding programs. This proportion is lower than what we observed in our collection, attesting to the important role that ICRISAT played in peanut germplasm sharing in African breeding programs. Germplasm exchanges between breeding programs were also important, attested by 9% of genotypes that were present more than once in the collection. However, when analyzing the genotyping results, we identified germplasms with the same names that were genotypically divergent. For example, this was the case for 55-437 which was nominated 5 times with 3 divergent versions, or for Fleur11 which was nominated 4 times with 2 divergent versions. Conversely, lines with different names could refer to the same or very closely related genotypes (pale yellow clusters in Fig. 3). For instance, ICG 12991 is a short duration, drought-tolerant, Spanish-type peanut germplasm line (Reg. no. GP-122, PI 639691) which was released in Malawi as “Baka” in 2001 and in Uganda as “Serenut 4T” in 2002. ICG 12991 and Baka were in close vicinity of the tree while Serenut 4T was more distant. Similarly, ICGV-SM 83708 (Reg. no. GP-68, P1 585000), an improved peanut germplasm, was released in 1990 as “CG 7' in Malawi, in 1991 as “MGV 4' in Zambia, and in 1998 as “Serenut 1R” in Uganda (Nigam et al. 1995; Deom et al. 2006; Okello et al. 2010; Tabe-Ojong et al. 2022). CG 7 and MGV 4 were close to each other on the tree while Serenut 1R was much more distant. These observations indicate that germplasm management in breeding programs is still challenging and the sources of errors are similar to what is observed in genebanks: redundant genotypes, seed mixture, and mislabeling (McClung et al. 2020). This also highlights the added value of marker-assisted germplasm management for accurate genotype identification in breeding programs.

Another remarkable result is the cluster of lines highlighted in pale green in Fig. 3 composed of Fleur11 (Ghana and Senegal versions) and several Chromosome Segment Substitution Lines (CSSLs) (12CS series) derived from a marker-assisted backcrossing program in Senegal using Fleur11 as recurrent parent and a synthetic tetraploid combining the genomes of the wild species *A. ipaensis* and *A. duranensis* (Fonceka et al. 2012). The CSSLs were shared with ICRISAT in 2013 and distributed through ICRISAT to some national programs in Africa including Zambia and Mali which nominated 4 lines each. Interestingly, 7 lines from Togo (with the suffix Tog-HG), 5 lines from ICRISAT (with the suffix ICGV-IS) nominated by Mali and Ghana, and 1 line from Uganda were also in the same cluster. This suggests either a renaming of the lines or lines that were derived from similar interspecific crosses. The first hypothesis illustrates the mechanisms for creating redundancy in breeding programs via lines renaming and sharing.

### Germplam collection composition and diversity in the breeding programs

Among the 1,049 genotypes assembled in this study, 769 (73%) belonged to the *fastigiata* subspecies, and 280 (27%) to the *hypogaea* subspecies, based on their position in the Ward clustering tree. Model-based structure analysis at K = 5 also supported the partitioning of the collection into 2 subspecies and 3 market types. The structure group #3 was represented by a limited number of typical Valencia varieties, like Acholi white, Numex 01, Numex 02, or Red Beauty. Many varieties that had significant Valencia ancestry were actually the result of an admixture between Valencia, Spanish, and/or Virginia (structure groups #2 and #1), like Kayoba variety from Zambia or Numex 03 and 04 from Ghana. The Spanish group was further divided into 3 groups. Likewise, high level of admixture was observed in some genotypes of this market type. Interestingly, the structure group #4 which was composed of germplasm from interspecific origin showed contributions to other genotypes that were known as having interspecific progenitors like Jaambar or Kom-Kom. Although genotypes that belong to *hypogaea* subspecies were more frequent in East and Southern Africa, we noted an under-representation of these subspecies when comparing the composition of our collection to other collections reported earlier and also used for core or mini-core collection development. Accessions from *hypogaea* subspecies represented approximately 46%, 35%, and 42% of the collection of 1,705 accessions developed by ICRISAT (Upadhyaya et al. 2002), of the USA collection of 831 accessions (Holbrook et al. 1993; Otyama et al. 2020) and of the China collection of 576 accessions (Jiang et al. 2010), respectively. It is worth noting that our collection was derived from lines nominated by breeders while the ones cited above were constructed to represent the diversity existing in genebanks. The under-representation of *hypogaea* subspecies in the germplasm managed by the breeding programs in Africa could be a result of a progressive shift of the breeding programs toward the use of short duration genotypes that are mostly found in *fastigiata* subspecies (Ferguson et al. 2004) to develop new varieties, as a result of shortening of the rainy seasons in East, Southern and West Africa countries (Camberlin et al. 2009; Salack et al. 2015). When considering market types, Valencia represented only 3.3% and was mostly nominated by breeding programs from East and Southern Africa.

Expected heterozygosity (He) is a reliable measurement of the genetic diversity of populations with finite size, such as the ones used in breeding programs, particularly when they are genotyped with thousands of SNP markers (Fu 2015). In our study, we first calculated He for all genotypes of each breeding program. We then recalculated He while removing the more closely related genotypes, as germplasm in breeding programs could be sister lines, to avoid bias of high relatedness. In both cases, He values ranged between 0.19 and 0.39, with Niger breeding program showing the lowest gene diversity and Zambia, Uganda, Malawi, and Ghana1 the highest ones. The low gene diversity in Niger breeding program is mainly due to the lack of lines belonging to the *hypogaea* subspecies (Fig. 4). An increase in He values was observed in Mali, Senegal, and Togo when removing closely related genotypes attesting a significant proportion of closely related material among the genotypes nominated by these programs. Comparing gene diversity between breeding program is not straightforward as He is sensitive to sample size, marker types, and number (Barrandeguy and García 2021). In our study, apart from Senegal and Uganda breeding programs, the number of genotypes was similar, easing the comparison between breeding programs. We also tried to compare the gene diversity of the 10 breeding programs in Africa with the ones available in published studies that have similar sample size and marker type and number. One such study analyzed the diversity of 96 lines from the Korean peanut collection using SNP markers (Nabi et al. 2021). Our comparison showed that, except for Niger, the gene diversity level in the breeding programs (0.25–0.39) is higher than what was observed in Korean peanut germplasm (0.22). The gene diversity in some breeding programs was similar (Senegal, Ghana2, Mozambique) or even higher (Malawi, Uganda, Togo, Zambia) to the one of a larger set of germplasm including the Korean germplasm and selected accessions of the US core-collection (Nabi et al. 2021), indicating a high level of genetic diversity in the breeding programs in Africa.

### The core collection is a highly valuable resource for breeding and gene discovery

In this study, we developed a core collection based on traits (breeder choices) and on diversity (genetic distance-based sampling). The core collection has higher gene diversity than the individual breeding programs, although its contribution to enlarging each breeding program genetic diversity is variable (Fig. 4). Core collection development has been reported in rice (reviewed by Raturi et al. 2022) and in many other important crop species (reviewed by Upadhyaya et al. 2006). In peanut, a Chinese mini-core collection has been extensively used for mapping traits related to plant, pod, and seed morphology and yield components traits (Jiang et al. 2014; Zhou et al. 2021) as well as for traits related to aflatoxin resistance (Ding et al. 2022). The peanut US mini-core collection was also used to identify QTLs for early and late leaf spot (Zhang et al. 2020) and seed size (Chu et al. 2019). Zou et al. (2021) combined the Korean collection and part of the US mini-core for mapping QTLs for seed aspect ratio. However, as most of the core-collections were developed to represent the diversity that exists in large genebank collections, they are much more adapted for trait discovery and prebreeding. Because our core collection has been mainly composed of varieties and breeding lines, we anticipate that it will be valuable for direct use by breeders in Africa, while being adapted for QTL mapping. Indeed, several elite lines with known characteristics for traits of agronomic and nutritional importance are part of the core collection. This is particularly the case for high oleic trait with lines ICGV 15017, ICGV15021, ICGV 15025, ICGV 15046, Schubert, Dogo-Chin4, and Numex-05 (Pandey et al., 2020; Burow et al. 2014; Dave Hoisington, pers. comm.), and late leaf spot and rust resistance for which 17 lines of the core collection harbor the well-known *A. cardenisii* chromosome segments on chromosomes A02 and A03 (Bertioli et al. 2021). Moreover, the core collection we developed has recently successfully been used for mapping QTLs for late leaf spot resistance (Oteng-Frimpong et al. 2023) and Groundnut Rosette Disease resistance (Achola et al. 2023).

Several lines in the core collection are already in advanced release pipelines as direct introductions in many breeding programs. Numerous crosses have been made and populations are being advanced that suit various product profiles and market segments. The availability of this core germplasm to national programs provides the much-needed resources for any breeding programs since groundnut and its wild relatives are not part of Annex 1 of the Multilateral System (MLS) of the International Treaty on Plant Genetic Resources for Food and Agriculture (ITPGRFA) thereby affecting its global movements (Tabe-Ojong et al. 2022). There is need for more nominations of new lines, landraces, and breeding lines for genotyping to further enrich this core collection diversity. In-depth pedigree information could provide more insights into the links between germplasm ancestry.

## Supplementary Material

jkad244_Supplementary_Data

## Data Availability

The genotyping data used in this study is available from the gigwa instance hosted by PeanutBase at https://peanutbase.org/gigwa under the “African_Lines_1049' project. All germplasms analyzed in this study are available upon request to the corresponding author. Supplemental material available at G3 online.
