# Supplementary material for: The groundnut improvement network for Africa (GINA) germplasm collection: a unique genetic resource for breeding and gene discovery
Source: G3 (Bethesda). 2023 Oct 24;14(1):jkad244. doi: 10.1093/g3journal/jkad244 (PMC10755195; doi:10.1093/g3journal/jkad244)
Supplement: jkad244_Supplementary_Data [file jkad244_supplementary_data.zip › Supplemental_Material_Legends_G3-2023-404499.docx]

# Supplemental Material

Figure S1: first principal plan of the principal component analysis of the African germplasm collection based on SNP data. For each of the seven clusters of closely related varieties, only one member was kept in the PCA analysis and other members of each cluster were projected as supplementary individuals.

Table S1: list of 1049 varieties of the African germplasm collection.
