# Supplementary figures and images for: The groundnut improvement network for Africa (GINA) germplasm collection: a unique genetic resource for breeding and gene discovery
Source: G3 (Bethesda). 2023 Oct 24;14(1):jkad244. doi: 10.1093/g3journal/jkad244 (PMC10755195; doi:10.1093/g3journal/jkad244)

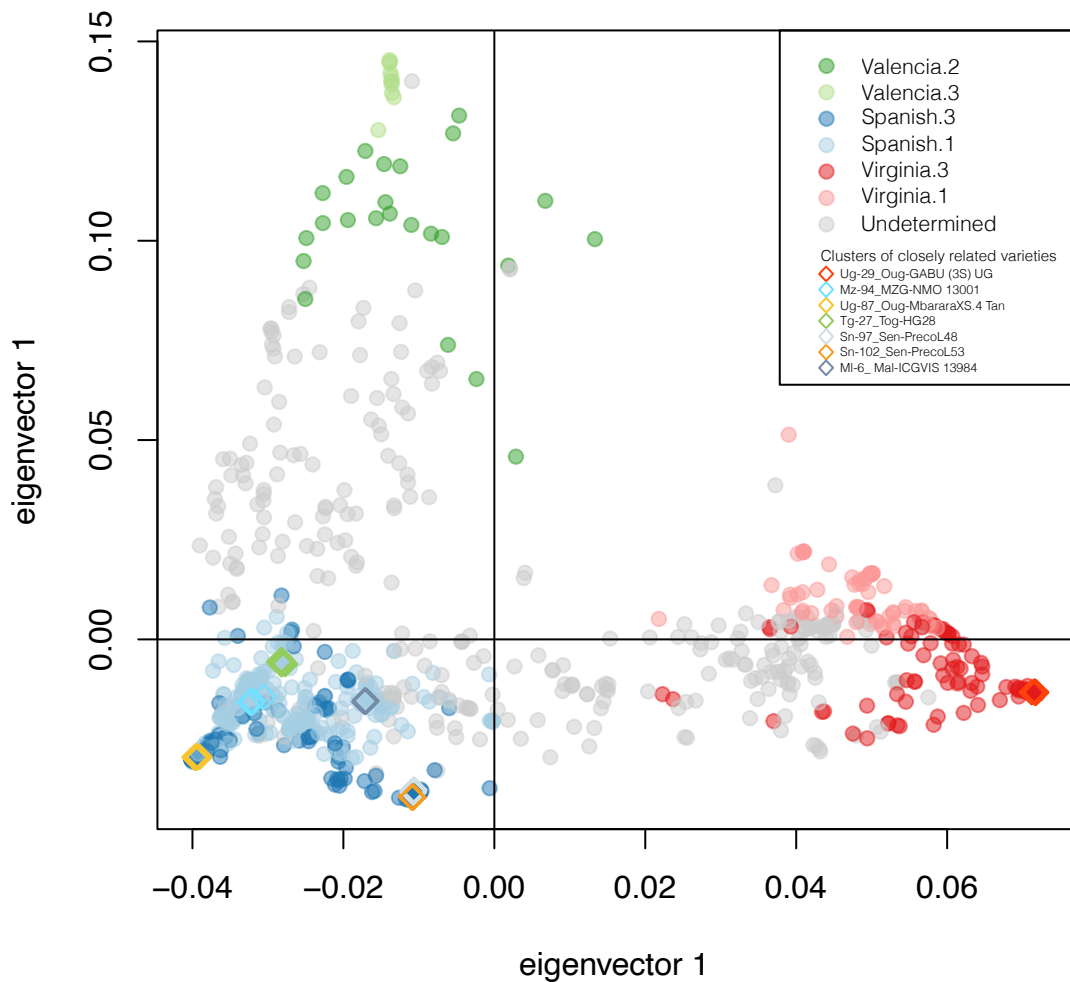

Supplement: jkad244_Supplementary_Data [file jkad244_supplementary_data.zip › Figure_S1_G3-2023-404499.pdf]
